# “Minho Technique” for Laparoscopic Radical Cystectomy with Intracorporeal Ileal Conduit

**DOI:** 10.5152/tud.2024.23230

**Published:** 2024-05-01

**Authors:** Andreia Cardoso, Sara Anacleto, Catarina Laranjo Tinoco, Ana Sofia Araújo, Mariana Capinha, Luís Borges Pinto, Aparício Coutinho, Catarina Tavares, Vera Marques, Paulo Mota, Miguel Mendes, Carlos Oliveira, João Pimentel Torres, Emanuel Carvalho-Dias

**Affiliations:** Department of Urology, Hospital de Braga, Braga, Portugal

**Keywords:** Ileal conduit, laparoscopy, radical cystectomy

## Abstract

**Objective:**

Radical cystectomy (RC) with ileal conduit (IC) remains a main treatment for muscle-invasive bladder cancer (MIBC). Laparoscopy in this multistage surgery is quite demanding, so laparoscopic RC (LRC) with intracorporeal IC (IIC) is a technically exceptional procedure. We aim to simplify it, demonstrating our technique, step-by-step.

We present a 4-port LRC with IIC and Bricker uretero-ileal anastomoses. The main difference is the immediate and complete posterior dissection, similar to the “Montsouris approach” for prostatectomy.

**Materials and Methods:**

A 70-year-old man with a 5 cm MIBC was subjected to our demonstrated procedure.

**Results:**

The postoperative period was uneventful. Diet and ambulation: 2 days. Single-J stents removal: 4 weeks. MIBC N0 was confirmed. At 24 months, the patient is well, without complications (namely hydronephrosis or disease recurrence).

**Conclusion:**

LRC with IIC is demanding and requires laparoscopic expertise. However, if performed in a standardized fashion, as demonstrated through this case, and considering our center's experience, it seems feasible and safe with 4-port and standard material without a significant operative time increase, nor oncological or functional compromise.

**Main Points**

- Starting laparoscopic radical cystectomy with an immediate and complete posterior plane dissection until the prostatic apex, similar to the “Montsouris” approach for radical prostatectomy, facilitates the entire procedure and possibly decreases the risk of rectal injury.
- Globally, for successful anastomoses, assure: wide lumen, mucosa apposition without strangulation, tension-free and water-tightness; preservation of tissue vascularization; adequate length and no twisting or angulation for ureters, mesentery, and ileum.
- Laparoscopic radical cystectomy with intracorporeal ileal conduit is technically demanding but feasible and safe, with 4-port and standard material, without operative time increase, nor oncological or functional compromise, if performed in a standardized, step-by-step fashion, as shown.

## Introduction

Radical cystectomy (RC) with urinary diversion (UD) remains the gold-standard treatment for muscle-invasive bladder cancer (MIBC). Historically, open RC was first performed in 1887,^1^ while almost 100 years later, laparoscopic RC (LRC)^2^ and intracorporeal ileal conduit (IIC)^3^ were reported. More recently, the introduction of robotics significantly increased minimally invasive approaches for RC.^4^ Nonetheless, minimally invasive RC (MIRC) remains demanding, regarding time consumption and complications.

Here, we demonstrate, step-by-step, our modified technique of LRC. The main alteration is that the surgery starts with an immediate “Montsouris approach”.^5^ We present and discuss our technique in a problem-solution manner, our tips and tricks, aiming to simplify LRC and IIC, and to demonstrate how it can be a simpler, faster, safer, and reproducible procedure (Supplementary Material: Video 1).

## Description of Technique

### Position and Laparoscopic Entry

The patient is positioned in forced Trendelenburg, and the first trocar (for the camera) is inserted 2 fingerbreadths above the umbilicus. For this, we lift the umbilicus with Kocher forceps, incise the skin, and then perforate all abdominal wall layers with a Kelly haemostat, and introduce the trocar without an awl. The pneumoperitoneum is set at 12 mm Hg. 

Under vision, the following trocars are inserted in a fan-arranged manner: a 10 mm trocar lateral to the left rectus muscle, 1 cm below umbilicus level; a 5 mm trocar parallel to this, on the right; and a 10 mm trocar in the left iliac fossa. If an additional assisting surgeon is available, to facilitate the procedure, a fifth trocar (5 mm) can be placed in the right iliac fossa.

### LRC – “Montsouris Approach”

The first step of the surgery is the suspension of the sigmoid colon to the left abdominal wall, using a percutaneous guided suture. After percutaneous insertion of a straightened needle in the abdomen, we place it through fatty epiploic appendices of the colon to secure it to the abdominal wall. We never place the suture through the colon wall, and we always maneuver the needle and adjust the tension under camera vision, so this step does not risk colon wall perforation.

The conventional approach for MIRC starts with the identification and isolation of the ureters, followed by a lateral peritoneotomy^6^ for superior vesicle pedicle control. This approach leads the bladder to drop posteriorly, which complicates posterior plane dissection, requiring additional maneuvers to lift the bladder, and increases the risk of rectal injury.

**Figure 1.**
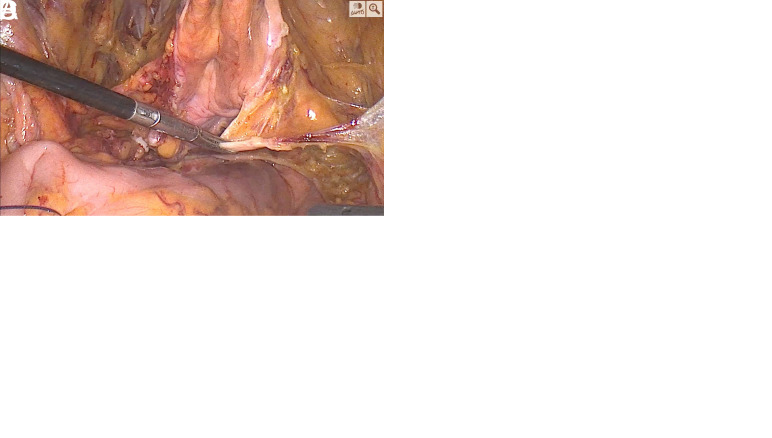
Surgical key steps in laparoscopic radical cystectomy. A – First posterior limited peritoneotomy. B – Posterior plane dissection. C – Superior vesical pedicle. D – Bladder natural suspension from remaining anterior attachments.

To overcome this, in our technique, after colon retro-suspension, we immediately perform a posterior dissection of seminal vesicles, vas deferens, and prostate, similar to the “Montsouris” radical prostatectomy.^5^ For this, we make a peritoneal transverse incision, 1-2 cm above the posterior peritoneal reflection, over the rectovesical pouch (Figure 1A), just enough to enable entire plane dissection of the seminal vesicles until the prostatic apex (Figure 1B). This facilitates the hardest and riskiest LRC step: the posterior dissection.

### LRC – Lateral Dissection

For lateral dissection, we start the peritoneotomy immediately lateral to the medial umbilical folds, and then we follow, from up to down, the obliterated umbilical artery until its origin in internal iliac artery. This maneuver quickly and safely leads us to the superior vesical artery origin, and allows its subsequent ligation (Figure 1C).

The ureters are easily identified beneath these lateral peritoneotomy lines. Thus, we are able to dissect and ligate them at the ureterovesical junctions with minimal mobilization.

To complete the lateral dissection, we proceed until the endopelvic fascia and puboprostatic ligaments.

After this total lateral exposure, we can safely and effortlessly ligate the inferior vesical pedicles. 

Performing this lateral approach after the posterior dissection allows us to perform a more easily nerve-sparing procedure, if desirable.

### LRC – Anterior Dissection

Finally, only anterior and apical attachments remain (Figure 1D). Now, the concern is to assure adequate urethral length preservation and avoid tumor spillage. For this, we release the urachus from the umbilicus, ligate Santorini's plexus, completely dissect the prostatic apex, preserving as much urethra as possible and ensuring oncological safety, and we apply a Hem-o-lok® to the Foley catheter before sectioning it. 

### Intracorporeal Ileal Conduit

**Figure 2.**
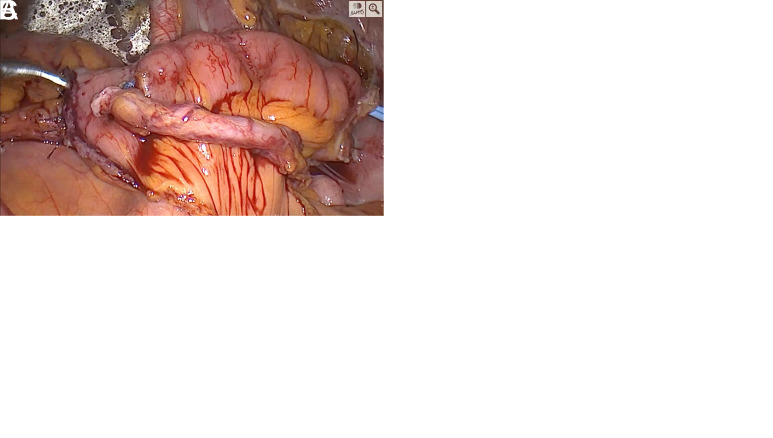
Surgical key steps in laparoscopic intracorporeal ileal conduit with Bricker uretero-ileal anastomosis. A – Ileum measurement with vessel loop. B – Created ileal conduit. C – Final image of intracorporeal ileal conduit with bilateral uretero-ileal anastomosis.

We identify the cecum, count 20 cm of distal ileum from the ileocecal valve, and another 15-20 cm for the ileal conduit(IC) (Figure 2A). At this step, it is particularly important not to section the mesentery too deeply. This decreases the probability of segmental ileal ischemic injury. Use the 60 mm suture length and triple staple line Endo-GIA®: it presents enough length for just one fire in all steps while avoiding excessive bleeding. Divide the ileum, perform a mechanical side-to-side ileo-ileal anastomosis, and shape the IC (Figure 2B).

Excessive mobility of the isolated IC can be solved through its fixation to the anterior abdominal wall, accomplishing great stability for the execution of uretero-ileal anastomoses (UIA). Left ureter transposition might not always be mandatory, but we perform it to maintain a more standard anatomy. 

For single-J stent placement, we puncture the abdomen with an abocath, introduce a guide-wire through it, and insert it into the ureter until the renal pelvis. Then, we place the single-J over it and leave it completely inside the abdomen. Finally, we introduce a forceps through a right-side trocar, then through the IC, perform the enterotomy in the desired place for the UIA, pass the forceps through this opening, grab the single-J, and position it throughout the IC.

We start with the left UIA due to ergonomic ease, and we prefer a Bricker UIA (Figure 2C). The stoma is created in a right-trocar incision. Lastly, we fill the IC with saline, remove the pneumoperitoneum, and, under vision, assure water-tight and tension-free anastomoses.

## Materials and Methods

A 70-year-old man, an active smoker, presented to the emergency department with macroscopic hematuria and clots. Urethrocystoscopy was suspicious for bladder cancer. A computed tomography (CT) scan revealed a 5 cm vesical mass, with suspicion of peri-vesical fatty tissue, prostate, and seminal vesicle invasion, but without distant metastasis. A transurethral resection was performed, and a high-grade urothelial MIBC with concurrent carcinoma in situ was retrieved. The patient started neoadjuvant chemotherapy (NAC) with gemcitabine and cisplatin. Re-evaluation by CT scan revealed a response to NAC, and patient was submitted to our 4-port LRC with IIC.

For our technique, only standard laparoscopic material is required: 3 10 mm and 1 5 mm trocars, a 10mm 30° laparoscope, harmonic scalpel, bipolar energy, a previously cut 15-20 cm length vessel loop, and a Laparoscopic Linear Cutter Stapler (60 mm suture length and triple staples line).

## Results

Operative time was 4 h 30. The postoperative period was uneventful. The patient started oral diet and ambulation on the second day. The drain was removed on day 4. The hospital discharge was at 8 days (due to antibiotics directed to the previous urine culture). Single-J stents were removed 4 weeks after surgery. The pathology report revealed an MIBC N0. After 24 months of follow-up, the patient is well, without complications (namely hydronephrosis), nor disease recurrence.

## Discussion

In the last years, MIRC grew worldwide.^4^ Despite the increase in OT at the beginning of the learning curve, and the concerns with potential complications and oncological results, these were surpassed, and MIRC advantages arose (pain, hospital stay, blood loss). Recent studies comparing minimally invasive with open RC demonstrate less perioperative morbidity, and even, potentially, a decrease in mortality.^7,8^

Still, intracorporeal UD remains a limitation due technical difficulty and increased OT. Nonetheless, we consider it the ultimate benefit of LRC, especially in the obese. Intracorporeal UD minimizes ureteral dissection and mobilization, thus preserving ureteral vascularization, one of the main factors for successful anastomoses, thus probably reducing UIA stricture risk.^9,10^

**Figure 3.**
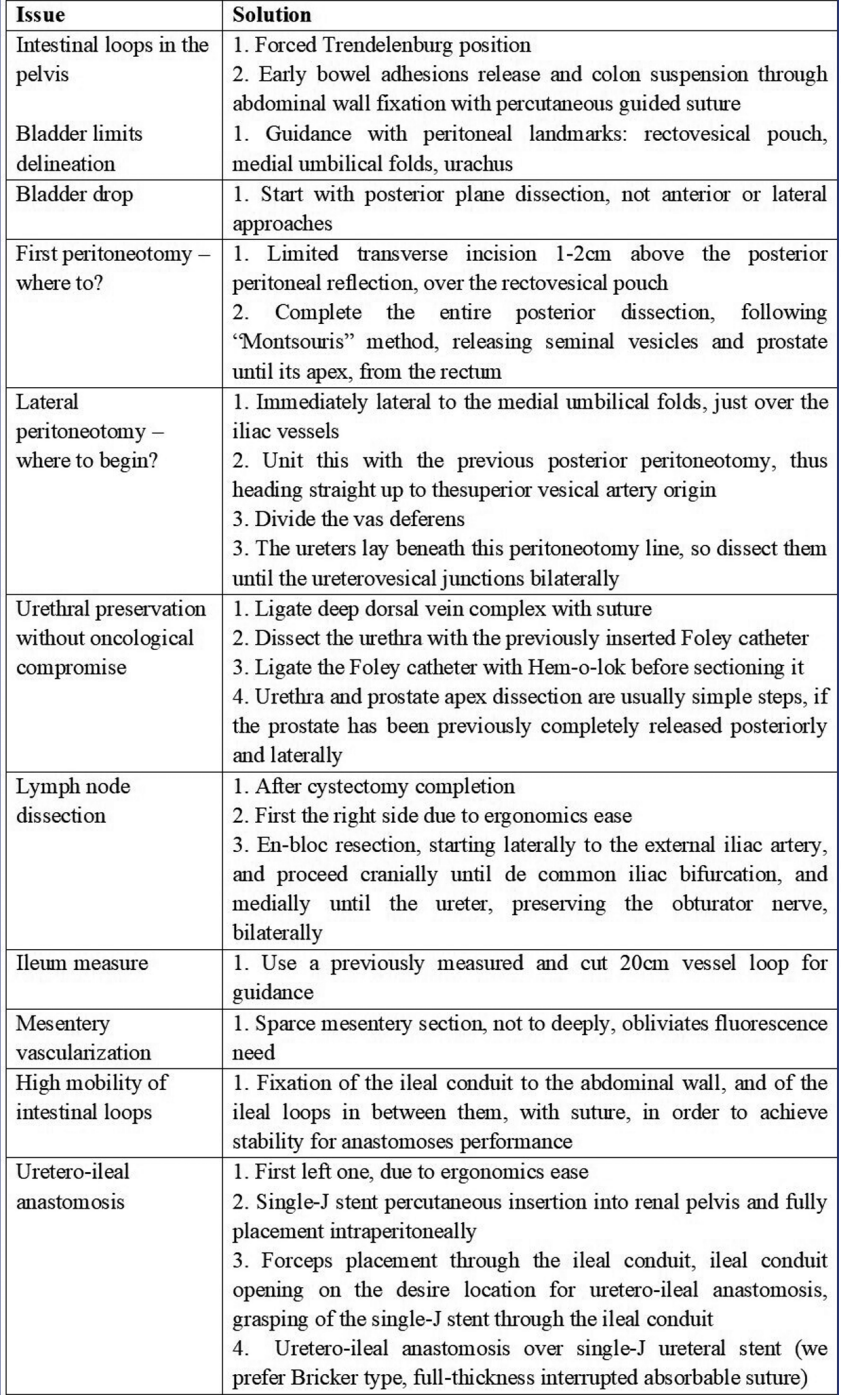
“Minho technique” for laparoscopic radical cystectomy with intracorporeal ileal conduit description step-by-step, in a problem-solution manner.

We believe our aforementioned tips simplify the extirpative phase of LRC with great advantages for novice laparoscopic/robotic surgeons (Figure 3). This technique probably decreases OT and allows for a safer dissection of seminal vesicles and prostate from the rectum, theoretically decreasing the risk of rectal injury. Also, our technique facilitates the execution of a nerve-sparing procedure.

Our goal was to demonstrate our technique, step by step, and in a standardized fashion, in order to make it more feasible for others who wish to try it. We have been using this technique for some years, with hundreds of LRCs performed in this way, even when the UD is extracorporeal. However, unfortunately, we do not maintain a regularly updated database about the outcomes of these patients. In spite of not being a case series review, but a technique description, we acknowledge as a limitation not having a large data set to report, which we expect to be able to present soon.

Lastly, we highlight some key points for UD: wide lumen, tension-free and water-tightness for all anastomoses; mucosa apposition without strangulation; tissue vascularization preservation; adequate length and no twisting or angulation of ureters, mesentery, and ileum. 

We recognize that LRC with IIC is demanding; however, from our experience, it seems feasible and safe with 4-port and standard material, without significant OT increase, nor oncological or functional compromise.
